# Improvement of Liver Transplantation Outcome by Heme Oxygenase-1-Transduced Bone Marrow Mesenchymal Stem Cells in Rats

**DOI:** 10.1155/2016/9235073

**Published:** 2016-01-05

**Authors:** Bin Wu, Hong-Li Song, Yang Yang, Ming-Li Yin, Bo-Ya Zhang, Yi Cao, Chong Dong, Zhong-Yang Shen

**Affiliations:** ^1^Department of Organ Transplantation, Tianjin First Central Hospital, Tianjin 300192, China; ^2^Tianjin Key Laboratory of Organ Transplantation, Tianjin 300192, China; ^3^Tianjin First Central Hospital Clinic Institute, Tianjin Medical University, Tianjin 300070, China

## Abstract

Bone marrow mesenchymal stem cells (BMMSCs) exert immunosuppressive activity in transplantation, and heme oxygenase-1 (HO-1) enhances their immunomodulatory effects. The aim of this study was to determine whether HO-1-transduced BMMSCs (HO-1/MSCs) improve rat liver transplantation (LTx) outcomes. Orthotopic LTx rejection models were treated with HO-1/MSCs, BMMSCs, HO-1, or normal saline, respectively. Our results showed a significant improvement in survival rates in the HO-1/BMMSCs group compared to the control groups. At all time points, liver function marker levels in the HO-1/MSCs group were significantly lower than in the other three groups; on POD 1, 7, and 14, the degree of rejection and apoptotic cells was significantly less in the HO-1/MSCs group than in the other three groups. Interleukin- (IL-) 10 and transforming growth factor-*β* levels were significantly increased, while IL-2, IL-6, IL-17, IL-23, tumor necrosis factor-*α*, and interferon-*γ* levels were significantly decreased in the HO-1/MSCs group when compared to the other groups. Splenocyte Tregs were significantly increased by HO-1/MSCs compared with controls on POD 3, 5, 7, 10, 14, and 28. Summarily, we provide evidence that HO-1/MSCs improved allogeneic LTx outcomes by attenuating inflammatory responses and acute cellular rejection, as well as enhanced immunomodulatory effects compared with BMMSCs.

## 1. Introduction 

Liver transplantation (LTx) is currently the only effective treatment for end-stage liver diseases, such as acute or chronic liver failure. However, the shortage of donor organs and issues of rejection and adverse reactions from immunosuppressants have hindered the use of Ltx. Immune rejection and ischemia-reperfusion injury after transplantation are two main reasons for loss of the graft [1]. Therefore, approaches to minimize rejection and induction of immune tolerance may be used to effectively address the above problems [2]. Clinical treatment of acute rejection (ACR) after LTx requires high doses of immunosuppressants. However, long-term use of immunosuppressive agents raises the risk of unfavorable side effects, such as nephrotoxicity, neurotoxicity, hypertension, posttransplant malignancy, and metabolic deterioration. Thus, safer and more effective strategies to attain immunosuppression are urgently needed.

Bone marrow mesenchymal stem cells (BMMSCs) are currently investigated in studies focused on transplantation immunity [3–5]. Because BMMSCs possess not only multidirectional differentiation potential and self-renewal capacity but also immunomodulatory activities, they are valuable in applications such as organ transplantation [6]. As a population of pluripotent stem cells from bone marrow, BMMSCs can be easily obtained and separated, as well as being cultivated and amplified* in vitro*. They may also be used* in vivo* without rejection and ethical obstacles. Based on the reasons above, we chose to use BMMSCs in our work. In recent years, BMMSCs have been shown to have low immunogenicity and therefore can suppress T cell-mediated immune rejection following organ transplantation [7], as well as participating in immunosuppression by influencing cytokine secretion from T cells and interacting with antigen presenting cells such as dendritic cells [8]. However, purified BMMSCs have been reported to have low activity after infusion* in vivo* [9], which was also noted in our earlier work.

Heme oxygenase (HO) is the rate-limiting enzyme in the degradation of heme to biliverdin and subsequently to bilirubin [10]. HO-1, an inducible isoform of HO, is a potent cytoprotective factor that has been shown to have anti-inflammatory, anti-ischemia-reperfusion injury, and antiapoptotic properties [11–13]. As an active regulatory factor involved in the control of immune tolerance after organ transplantation [14], HO-1 can increase the immunomodulatory effects of T regulatory cells (Tregs) by promoting the secretion of interleukin- (IL-) 10 and transforming growth factor- (TGF-) *β* to increase tolerance to grafts in recipients [15]. HO-1 also has been shown to reduce the apoptosis of BMMSCs under conditions of hypoxia and oxidative stress [16]. At present, genetic engineering can be used to effectively modify protein expression of BMMSCs [17]. Recent studies have found that transduction of HO-1 into BMMSCs improved their transformation ability [18], affected their immunomodulatory effects [19] and antioxidant capacity, and enhanced the strength and durability of their activities. In our previous studies, we found that HO-1-transduced BMMSCs (HO-1/MSCs) had improved immunoregulatory effects on lymphocytes* in vitro* compared with unmodified BMMSCs [20]. Thus, in this study, we verified those* in vitro* results using an orthotopic LTx rejection model to further determine whether HO-1/MSCs can improve outcomes of LTx in rats.

## 2. Materials and Methods

### 2.1. Animals and Ethics

Specific pathogen-free (SPF) experimental animals were obtained from the Vital River Company (Beijing, China). Inbred adult male Lewis rats (220–250 g, 8–10 weeks old) were LTx donors, and inbred adult male Brown Norway (BN) rats (220–250 g, 8–10 weeks old) were recipients. BMMSCs were extracted from inbred adult male BN rats (100–120 g, 4–5 weeks old). Before testing, all rats were housed individually for 3 days in standard animal facilities on a 12 h light/dark cycle and provided with commercially available chow and tap water* ad libitum*. All experimental procedures were carried out in accordance with the Guide for the Care and Use of Laboratory Animals of the National Institutes of Health (NIH publication 86-23, revised 1985). All protocols were approved by the Animal Care and Research Committee of Tianjin First Central Hospital, Tianjin, China (Permit number: E20130825-003A). Surgeries were performed using chloral hydrate anesthesia, and all efforts were made to minimize animal suffering.

### 2.2. Isolation and Characterization of BMMSCs

Using the method described by Pittenger et al. [21], BMMSCs were isolated from the femur and tibia of male BN rats (100–120 g, 4-5 weeks old) after sacrifice by cervical dislocation. After lysis of red blood cells using 0.1 mol/L NH_4_Cl, the remaining cells were washed, resuspended, and cultured for 4 weeks at 37°C with 5% CO_2_ in Dulbecco's modified Eagle medium (DMEM)/F12 (Gibco, Carlsbad, CA, USA) containing 100 U/mL penicillin, 100 mg/mL streptomycin, and 15% fetal bovine serum. The culture medium was changed every 72 h. After reaching 80% confluency, cells at the third passage were trypsinized, washed, centrifuged, and resuspended at 1 × 10^7^ cells/mL in phosphate-buffered saline (PBS). BMMSCs were labeled with antibodies against CD29, CD90, CD34, CD45, RT1A, and RT1B (Santa Cruz Biotechnology, Dallas, TX, USA) for flow cytometric analysis (BD FACSAria III, Franklin Lakes, NJ, USA). BMMSCs were also confirmed by light microscopy to be adherent to plastic and spindle-shaped.

### 2.3. Transduction of BMMSCs with HO-1-Bearing Recombinant Adenovirus

Adenovirus/HO-1 (HO-1) obtained from Shanghai Genechem Limited Company (Shanghai, China) was diluted to 10 pfu/cell with complete culture solution and used to replace the original medium of the BMMSC cultures. After 6 to 8 h, the HO-1 culture solution was exchanged with complete culture solution for continued cultivation of the BMMSCs. After 48 h, the infection efficiency was observed under a fluorescence microscope. Molecular biological features of HO-1/MSCs were assessed by flow cytometry.

### 2.4. Surgical Procedures and Experimental Protocol

An orthotopic LTx rejection model was performed with Lewis donor rats and BN recipient rats in a sterile field under general anesthesia using 5% chloral hydrate (10 mL/kg). Food was withheld from both donor and recipient animals for 12 h prior to surgery, and differences between their weights were not greater than 10 g. The two-cuff technique for LTx was used by a single operator [22]. The donor livers were perfused via the portal vein (PV) with 4°C lactated Ringer's solution containing heparin sodium (50 U/mL). The harvested graft was preserved in a bath of lactated Ringer's solution at 4°C. The cuff was slipped over the PV using microforceps, and the distal end of the vein was everted over the cuff and secured with a circumferential 5-0 silk ligature. The same procedure was performed for the infrahepatic vena cava (IHVC) cuff preparation. After the recipient liver was removed, the donor liver was placed orthotopically in the abdominal cavity of the recipient. The suprahepatic vena cava (SHVC) was anastomosed end to end using a continuous 7-0 nylon suture. The cuff anastomosis of the PV and IHVC was then performed. The graft was reperfused by opening the PV, IHVC, and SHVC in turn. The bile duct was connected by telescoping a tube in the bile duct of the donor into that of the recipient. Experimental animals were divided into four groups, which received HO-1/MSCs, BMMSCs, HO-1, or normal saline. Rats of the treatment group received 5 × 10^6^ HO-1/MSCs through the superficial dorsal veins of the penis immediately after the surgery, while the control groups were given 5 × 10^6^ BMMSCs, 5 × 10^7^ HO-1, or the equivalent volume of normal saline. Five animals per group were euthanized on postoperative day (POD) 1, 3, 5, 7, 10, 14, or 28.

### 2.5. Survival Rates of Recipients

Recipient survival rates and clinical manifestations were observed in five animals per group. Humane endpoints were used for moribund animals after surgery, especially in the survival study. All animals meeting the humane endpoint criteria were euthanized by intraperitoneal injection of excess chloral hydrate when there was no unexpected death.

### 2.6. Liver Function Assay

Recipient serum was obtained from peripheral blood. Levels of alanine transaminase (ALT), aspartate aminotransferase (AST), and total bilirubin (TBIL) were measured using an automatic biochemical analyzer (Olympus Au640, Tokyo, Japan).

### 2.7. Histopathological Analysis

After fixation in 10% formalin, hepatic tissues were embedded in paraffin and cut into 5 *μ*m thick sections, followed by staining with hematoxylin and eosin (H&E). Pathological changes and the extent of rejection were evaluated under a light microscope. ACR was classified according to the Banff criteria [23].

### 2.8. Apoptosis Assay

Terminal deoxynucleotidyl transferase- (TdT-) mediated dUTP nick end-labeling (TUNEL) staining was performed on paraffin-embedded tissue sections using the In Situ Cell Death Detection Kit (Roche Biochemicals, Mannheim, Germany), as instructed by the manufacturer. Tdt was not used in the negative controls, and deoxyribonuclease was used for the positive controls. Apoptotic nuclei would appear brown, while the staining of the cytoplasm would generally be light or absent. The slides were reviewed in a blinded fashion, and positive cells were counted in 10 randomly chosen fields under a light microscope (200x).

### 2.9. Enzyme-Linked Immunosorbent Assay (ELISA)

Serum was obtained from peripheral blood of recipients. Using ELISA, serum concentrations of cytokines related to inflammatory responses, T helper (Th)1/Th2, and Th17/Tregs were assayed at the same time points after LTx. Interleukin- (IL-) 10, TGF-*β*, IL-2, IL-6, IL-17, IL-23, tumor necrosis factor- (TNF-) *α*, and interferon- (IFN-) *γ* concentrations were measured using ELISA kits (Santa Cruz Biotechnology) according to the manufacturer's instructions.

### 2.10. Flow Cytometry

Lymphocytes were isolated from recipient spleens. Aliquots of 1 × 10^7^ cells were resuspended in 0.1 mL PBS and labeled with antibodies specific for CD4, CD25, and Foxp3 (eBioscience, San Diego, CA, USA) or their isotype-matched control antibodies for analysis by flow cytometry (BD FACSAria III). The anti-CD4 antibody was conjugated to fluorescein isothiocyanate (FITC), and the isotype-matched antibody was mouse immunoglobulin G (IgG)2a K-FITC. The anti-CD25 antibody was conjugated to phycoerythrin (PE), and the isotype-matched antibody was mouse IgG1 K-PE. The anti-Foxp3 antibody was conjugated to PerCP-Cyanine5.5, and the isotype-matched antibody was rat IgG2a K-PerCP-Cyanine5.5.

### 2.11. Statistical Analysis

Results are expressed as the mean ± standard deviation (SD) and compared by one-way analysis of variance. Kaplan-Meier analysis was used to compare survival rates of recipients, and log-rank testing was used to determine significant differences between groups. All statistical analyses were performed using SPSS statistical software, version 17.0 (SPSS GmbH, Munich, Germany), with *P* ≤ 0.05 considered statistically significant.

## 3. Results

### 3.1. Culture and Identification of BMMSCs

**Figure 1:**
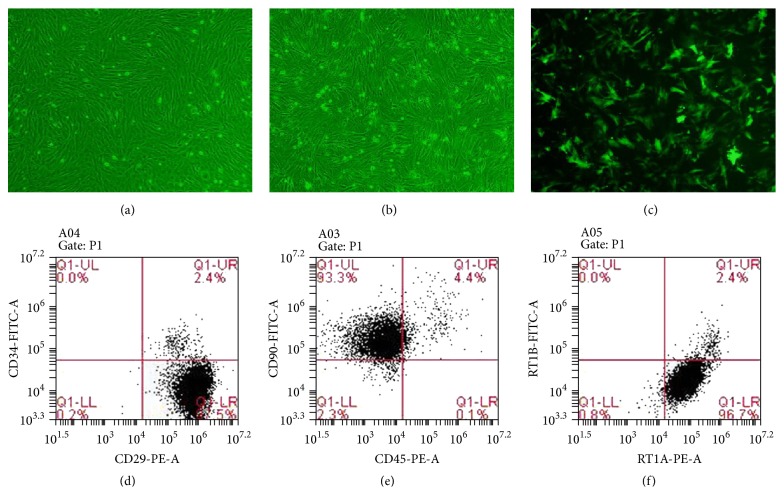
Morphological (100x) and flow cytometric analysis of BMMSCs. (a) Morphology of third-passage BMMSCs. (b) Morphology of BMMSCs after transduction with HO-1 in a bright field. BMMSCs exhibited spindle-shaped morphology and were arranged in whorls when transfected with HO-1. (c) Morphology of BMMSCs after transduction with HO-1 in a fluorescent field. Over 85% of BMMSCs after transduction with HO-1 emitted green fluorescence. (d) The proportion of CD29-positive and CD34-negative cells was 97.5%. (e) The proportion of CD90-positive and CD45-negative cells was 93.3%. (f) The proportion of RT1A-positive and RT1B-negative cells was 96.7%.

Cells isolated from rat bone marrow were confirmed as BMMSCs based on their spindle-shaped morphology, adherence to plastic, and phenotypic characterization by flow cytometry (Figure 1(a)). Surface marker detection of Ad/HO-1/BMMSCs showed that they were positive for CD29, CD90, and RT1A and negative for CD34, CD45, and RT1B (Figures 1(d), 1(e), and 1(f)).

### 3.2. Generation of HO-1/MSCs

At 48 h after HO-1 transduction, BMMSCs were observed by fluorescence microscopy, which showed that the infection efficiency was about 85% (Figures 1(b) and 1(c)) and indicated that HO-1/MSCs were generated successfully.

### 3.3. Improvement of Clinical Manifestations and Recipient Survival Rates

**Figure 2:**
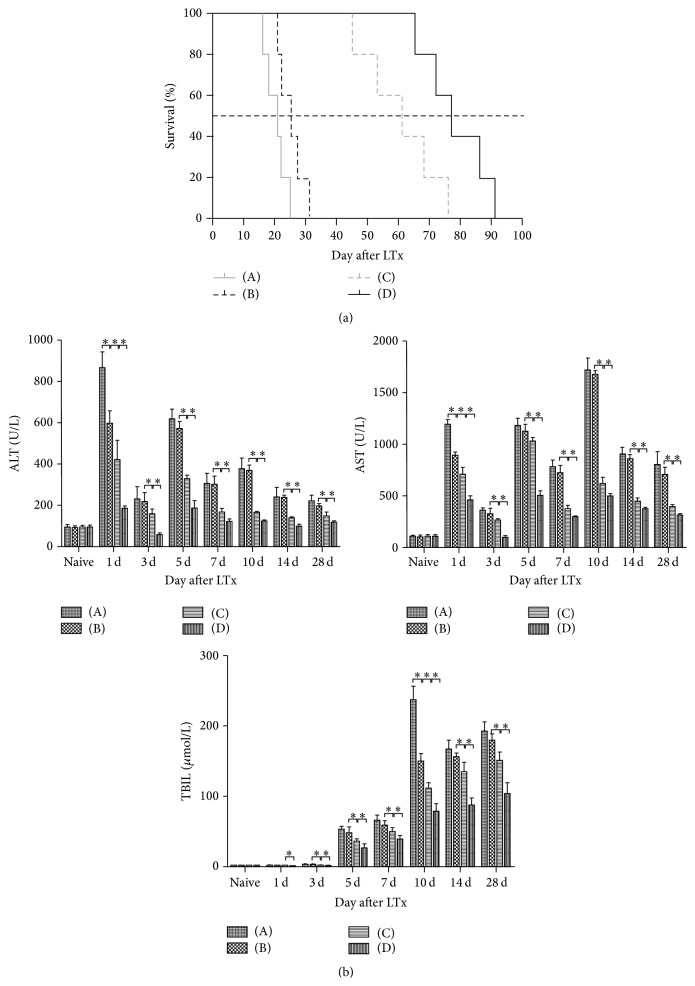
Kaplan-Meier survival curve of recipients. (A) Normal saline group; (B) HO-1 group; (C) BMMSCs group; (D) HO-1/MSCs group. (a) Median recipient survival times were 77, 61, 25, and 21 days in the HO-1/MSCs group, BMMSCs group, HO-1, and normal saline groups, respectively (*n* = 5 per group). Survival rates were significantly improved in the HO-1/MSCs treatment group compared with the three control groups (*P* < 0.05). (b) Levels of ALT, AST, and TBIL were significantly lower in the HO-1/MSCs group compared with control groups at all time points (^*∗*^ *P* < 0.05).

From POD 3 to 5, recipients in the HO-1 and normal saline groups had poor appetite, decreased activity, untidy and lusterless hair, jaundice, and lethargy. Most of them died within one month. However, most of the recipients in groups treated with HO-1/MSCs and BMMSCs survived for more than two months and were more active and responded more quickly to stimulation. The median recipient survival times were 77, 61, 25, and 21 days in the HO-1/MSCs, BMMSCs, HO-1, and normal saline groups, respectively. The survival time of the HO-1/MSCs group was significantly different from that of the other groups (*P* < 0.05), and that of the BMMSCs group was significantly different from the HO-1 and normal saline groups (61 versus 25 versus 21 days, *P* < 0.05; Figure 2(a)). Thus, HO-1/MSCs significantly improved the recipient survival rates.

### 3.4. Serum Levels of Markers of Recipient Liver Function

Within 7 days of LTx, levels of ALT and AST in all groups decreased at first, then increased, and finally decreased again. The level of TBIL in the normal saline and HO-1 groups increased steadily between POD 5 and 7, increased sharply on POD 10, and then decreased significantly but remained at a relatively high level. During that time, TBIL in the HO-1/MSCs and BMMSCs groups increased steadily to the maximum on POD 28, except for a slight decrease on POD 7. At all time points liver function marker levels in the HO-1/MSCs group were lower than in the other three groups (*P* < 0.05); those in the BMMSCs group were also significantly different when compared to the HO-1 and normal saline groups (*P* < 0.05; Figure 2(b)).

### 3.5. Histopathological Analysis and Grading of ACR in Hepatic Grafts

**Figure 3:**
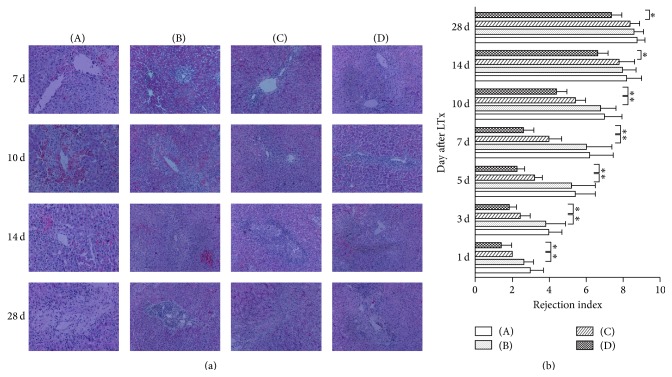
Histological sections of the rat liver and grading of ACR after LTx. (A) Normal saline group; (B) HO-1 group; (C) BMMSCs group; (D) HO-1/MSCs group. (H&E staining, 100x). (a) In the normal saline and HO-1 groups, ACR grading on day 7 was moderate to severe, with abundant mixed lymphocytes in the portal area, interlobular bile duct inflammation and damage, inflammatory cell infiltration in the vein area, and necrosis of liver cells. In the BMMSCs group, ACR grading was mild until day 7, was aggravated sharply after day 7, and was moderate to severe on day 14. ACR was attenuated in the HO-1/MSCs group and decreased at all time points, compared to control groups (^*∗*^ *P* < 0.05). (b) Histogram of ACR grading (*n* = 5 in each group, the HO-1-transduced BMMSCs group compared to control groups). Data are expressed as the mean ± SD (^*∗*^ *P* < 0.05).

ACR was classified by the grade of inflammatory infiltrate in the portal space, around biliary ducts and in vessel walls [19]. To analyze the effects of treatment with normal saline, HO-1, BMMSCs, and HO-1/MSCs on ACR of hepatic grafts in allogeneically transplanted animals, we assessed the histopathological grade of ACR at each time point after LTx. The animals treated with normal saline and HO-1 showed a gradual increase in acute rejection, with moderate or severe acute rejection observed on POD 5 and 7. Animals in both the BMMSCs and HO-1/MSCs treatment group showed reduced ACR to different degrees. The pathology of the HO-1/MSCs treatment group was characterized by mild rejection within POD 7, and the degree of rejection increased progressively thereafter; however, the degree of rejection in the HO-1/MSCs group was less than in the other three groups (*P* < 0.05), and that in the BMMSCs group was less than in the HO-1 and normal saline groups, at all time points (*P* < 0.05; Figure 3).

### 3.6. Evaluation of Apoptosis

**Figure 4:**
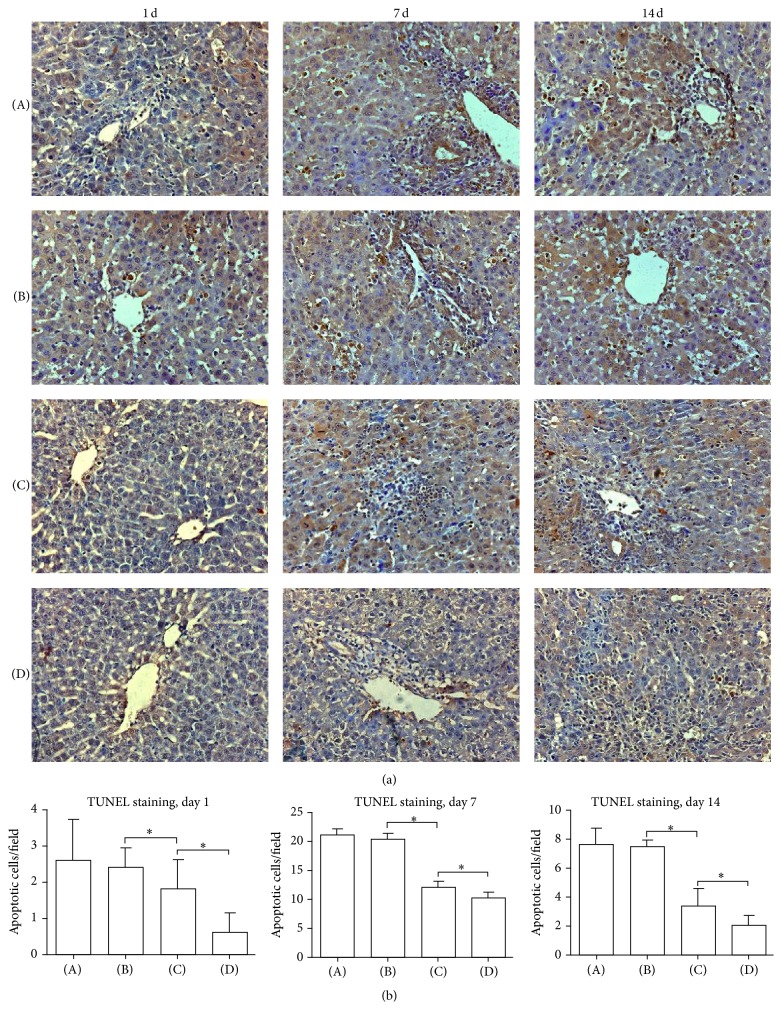
Apoptotic cells in liver grafts. (A) Normal saline group; (B) HO-1 group; (C) BMMSCs group; (D) HO-1/MSCs group. (a) Histological sections from rats of four groups at each time point were subjected to the TUNEL assay (200x). (b) Histogram showing the percentage of positive apoptotic signals in each group on days 1, 7, and 14. Apoptotic cell numbers in the HO-1/MSCs group were significantly lower compared with control groups on POD 1, 7, and 14, respectively (^*∗*^ *P* < 0.05).

ACR is associated with increased apoptosis in the graft. Using TUNEL staining, we evaluated apoptotic cells in the hepatic tissue after LTx (Figure 4(a)). On POD 1, 7, and 14, there were fewer apoptotic cells in the HO-1/MSCs group than in the other three groups (*P* < 0.05), and there were fewer apoptotic cells in the BMMSCs group than in the normal saline and HO-1 groups (*P* < 0.05). Treatment with HO-1/MSCs significantly reduced TUNEL-positive apoptotic cells in hepatic tissue at each time point (Figure 4(b)).

### 3.7. Recipient Serum Cytokine Concentrations

**Figure 5:**
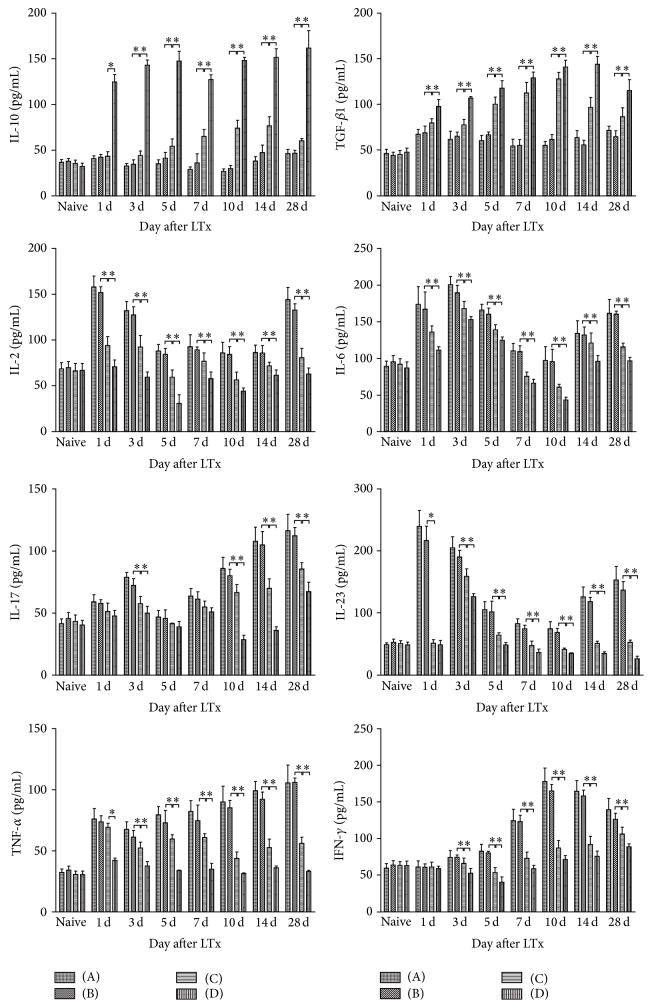
IL-10, TGF-*β*, IL-2, IL-6, IL-17, IL-23, TNF-*α*, and IFN-*γ* concentrations in recipient serum. (A) Normal saline group; (B) HO-1 group; (C) BMMSCs group; (D) HO-1/MSCs group. Cytokine concentrations in the recipient serum were measured by ELISA on days 1, 3, 5, 7, 10, 14, and 28 after transplantation (*n* = 5 in each group). Data are expressed as the mean ± SD (^*∗*^ *P* < 0.05). Levels of IL-10 and TGF-*β* were significantly higher, while levels of IL-2, IL-6, IL-17, IL-23, TNF-*α*, and IFN-*γ* were significantly lower in the HO-1/MSCs group than those of control groups.

ELISAs were performed to assay the serum concentrations of cytokines related to inflammatory responses and differentiation of T cells, such as Th1/Th2 and Th17/Tregs (Figure 5). The HO-1 and normal saline treatment groups showed rising trends in concentrations of IL-2, TNF-*α*, IFN-*γ*, IL-6, IL-17, and IL-23, and these levels were significantly increased compared with the BMMSCs and HO-1/MSCs group. Serum IL-10 and TGF-*β* concentrations in both the HO-1 and normal saline treatment groups also decreased rapidly. The decreases in IL-2, IL-6, and IL-17 levels were much lower in the BMMSCs treatment group compared with the allogeneic control group; however, their concentrations sharply increased on POD 10 compared with other time points. Concentrations of IL-23, TNF-*α*, and IFN-*γ* exhibited a relative small decrease in the BMMSCs treatment group, while those of IL-10 and TGF-*β* continuously increased. As for the HO-1/MSCs treatment group, IL-23, TNF-*α*, and IFN-*γ* exhibited relatively small decreases in concentration, and levels of IL-6, IL-17, and IL-2 demonstrated a downward trend until POD 10 and an upward trend thereafter; meanwhile, IL-10 and TGF-*β* concentrations continuously increased at all time points.

### 3.8. Proportion of Recipient Spleen Tregs

**Figure 6:**
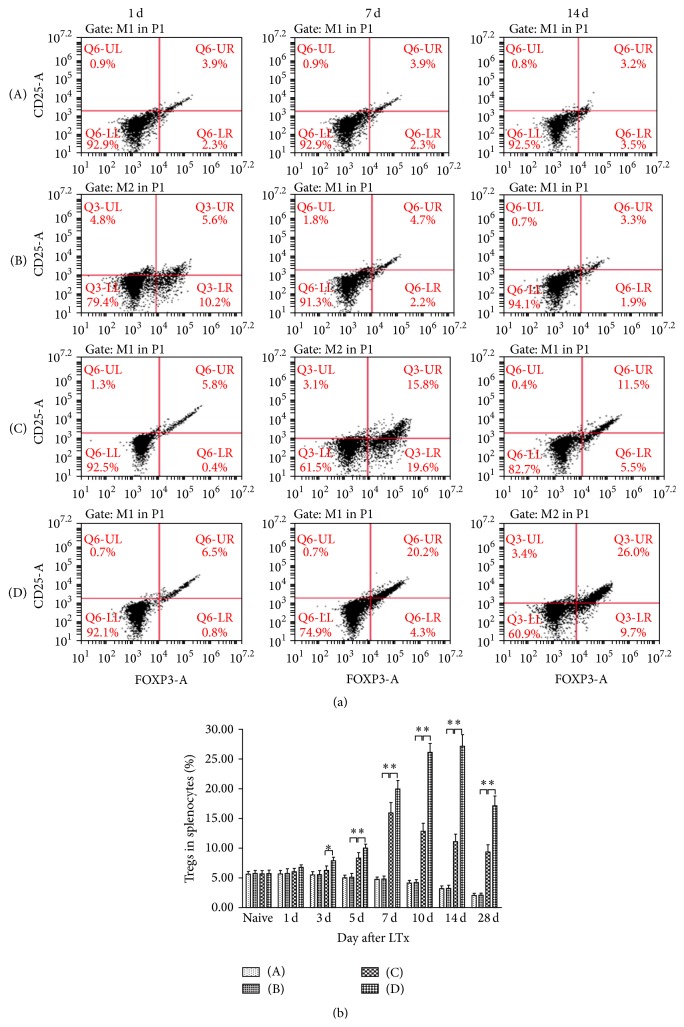
Proportion of Tregs in recipient splenocytes. (A) Normal saline group; (B) HO-1 group; (C) BMMSCs group; (D) HO-1/MSCs group. Percentages of CD4+CD25+Foxp3+ cells in recipient spleens were measured by flow cytometry. (a) Scatter plots on POD 1, 7, and 14 showing that the percentage of Tregs was higher in HO-1/MSC-treated rats than in the control groups. (b) Histogram showing percentages of Tregs on POD 3, 5, 7, 10, 14, and 28. The number of Tregs in the HO-1/MSCs group first increased and then decreased but peaked on POD 14 and were significantly higher than those of other groups on POD 3, 5, 7, 10, 14, and 28 (^*∗*^ *P* < 0.05).

Using flow cytometry, CD4+CD25+Foxp3+Tregs were assayed at various time points after LTx (Figure 6). The proportion of Tregs in splenocytes in the normal saline and HO-1 groups gradually decreased over time, with no significant difference between them. Levels of Tregs in the BMMSCs group first increased and then decreased, reaching the peak on POD 7 and showing significantly higher percentages than those of the normal saline group on POD 5, 7, 10, 14, and 28 (*P* < 0.05). Levels of Tregs in the HO-1/MSCs group also first increased and then decreased; however, they peaked on POD 14 and showed significantly higher proportions than those of other groups on POD 3, 5, 7, 10, 14, and 28 (*P* < 0.05).

## 4. Discussion

LTx is an effective treatment for end-stage liver diseases, but rejection and ischemia-reperfusion injury can affect survival in LTx recipients and mainly account for transplantation failures. Although the use of immunosuppressive drugs can mitigate immune rejection, their long-term application can also result in high costs and side effects, such as immune deficiency and tumor susceptibility. Induction of tolerance, a long-term and specific state of anergy of the immune system, in graft recipients should be achieved while maintaining their ability to respond to other foreign antigens. Thus, inducing a lasting and stable tolerance without drugs is an ideal goal [24]. Research studies worldwide have shown that BMMSCs and HO-1 not only exert independent immune protective effects in LTx but HO-1 also can help BMMSCs to protect grafts [12, 13, 25]. Therefore, this study was designed to observe the immune regulatory effects of HO-1/MSCs in LTx and explore the potential related mechanism.

In the current study, an orthotopic liver LTx rejection model was established with major histocompatibility complex- (MHC-) disparate rat strains (Lewis to BN). Histopathological analyses showed that the ACR reaction gradually increased in the normal saline group, with moderate to severe ACR occurring on day 7. The trend in the HO-1 group was nearly the same as that in the normal saline group, in which the degree of ACR was slight and not obviously different, suggesting that the effect of infusing pure HO-1 was not ideal. BMMSCs could attenuate the allograft rejection until day 7. From day 10, however, the improvement was attenuated, likely due to the reduction in numbers of BMMSCs and their diminished effects* in vivo*. Not only did HO-1/MSCs markedly attenuate the allograft rejection, but the improvement was still obvious after day 7, indicating that HO-1 could enhance and prolong the duration of the immune activities of BMMSCs* in vivo*, thus resulting in better immunomodulatory effects. Furthermore, levels of ALT, AST, and TBIL were significantly lower in the HO-1/MSCs group than control groups at all time points. Therefore, these results further confirmed the strong immune regulatory and enhanced protective effects of HO-1/MSCs on liver cells.

The pathogenesis of organ transplant rejection has been shown to be mainly mediated by T cell immune responses [26]. Functionally, T lymphocytes can be divided into CD4+ Th cells, CD8+ killer T cells (cytotoxic T cells), and suppressor T cells. Th cells can secrete a variety of cytokines, which are classified as Th0, Th1, Th2, Th3, or Th17, to regulate immune responses and play an important role in transplantation immunity [27]. Th1 cells can trigger allogenic transplant rejection by promoting the generation of alloantigen specific cytotoxic T cells and delayed-type hypersensitivity, while Th2 cells exert opposite effects. Therefore, the dynamic Th1/Th2 balance plays an important role in immune tolerance, and the shift from Th1 to Th2 type cells is one mechanism of generating tolerance [28]. As IL-2 and IFN-*γ* are Th1 cytokines and IL-10 is a Th2 cytokine, the IL-2/IL-10 ratio closely reflects the rate of rejection and induction of tolerance [29]. The dynamic Th17/Treg balance also is important in immune tolerance. IL-6, IL-17, IL-23, and TGF-*β* are Th17/Treg-related cytokines, which determine the transition of T cells into inflammatory Th17 or immune Tregs [30]. As a proinflammatory cytokine, TNF-*α* is closely related to immunoregulation, cell apoptosis, and T cell proliferation, and it has been positively correlated with the degree of graft rejection [31]. In this study, levels of IL-10 and TGF-*β* were significantly higher, while those of IL-2, IL-6, IL-17, IL-23, TNF-*α*, and IFN-*γ* were significantly lower, in the HO-1/MSCs group compared with control groups, which further confirmed the strong immune regulatory effects of HO-1/MSCs.

Tregs (CD4+CD25+Foxp3+ T cells) exert negative immune regulation and account for approximately 5–10% of peripheral CD4+T cells. By inhibiting the activation and proliferation of other immune effector cells, Tregs play an important role in the induction of immune tolerance to transplanted tissue. Studies to date have implicated the following mechanisms for Treg-mediated inhibition of other immune effector cells: inhibiting IL-2 production by close contact among cells; preventing antigen presenting cells from expressing stimulus molecules and preventing dendritic cells from maturing; destroying target cells via the granular enzyme/perforin death pathways; consuming local IL-2 to cause loss of stimulatory signals to effector T cells; and inducing production of inhibitory cytokines such as TGF-*β*, IL-10, and IL-35 [32]. In this study, the proportion of Tregs was significantly higher in the HO-1/MSCs group compared with control groups on POD 3, 5, 7, 10, 14, and 28. Therefore, we concluded that inducing production of Tregs is just one mechanism by which HO-1/MSCs can exert their immunosuppressive effects, and the selective inhibition of Tregs is led by the combined effects of various inhibitory mechanisms.

In summary, in this study, compared with BMMSCs, HO-1/MSCs improved the pathology of the transplanted liver, reduce cell apoptosis, improved liver functions, decreased levels of Th1 and Th17 cytokines, and promoted the generation of Tregs. Therefore, we reasoned that HO-1/MSCs could improve the outcome of allogeneic LTx by attenuating the inflammatory response and ACR, with better and more prolonged effects compared to BMMSCs. However, further studies exploring mechanism are needed to fully elucidate how HO-1 helps BMMSCs to attenuate the inflammatory response and ACR. In addition studies using higher doses of HO-1 are required. We believe that these findings provide a potentially new and effective strategy for suppressing ACR and improving outcomes after LTx.

## Abbreviations

BMMSCs: Bone marrow mesenchymal stem cells
BN: Brown Norway
ELISA: Enzyme-linked immunosorbent assay
GFP: Green fluorescent protein
HO-1: Heme oxygenase-1
LTx: Liver transplantation
ACR: Acute cellular rejection
POD: Postoperative days
IFN-*γ*: Interferon-*γ*
IL-2: Interleukin-2
IL-6: Interleukin-6
IL-10: Interleukin-10
IL-17: Interleukin-17
IL-23: Interleukin-23
PBS: Phosphate-buffered saline
TGF-*β*: Transforming growth factor-*β*
TNF-*α*: Tumor necrosis factor-*α*
Tregs: T regulatory cells
TUNEL: Terminal deoxynucleotidyl transferase-mediated dUTP nick end-labeling.
